# The Convergent Immunopathogenesis of Cigarette Smoke Exposure: From Oxidative Stress to Epigenetic Reprogramming in Chronic Disease

**DOI:** 10.3390/ijms27010187

**Published:** 2025-12-24

**Authors:** Aysen Kutan Fenercioglu, Hafize Uzun, Durisehvar Ozer Unal

**Affiliations:** 1 Department of Family Medicine, Cerrahpasa Medical Faculty, Istanbul University-Cerrahpasa, Istanbul 34098, Türkiye; 2 Department of Medical Biochemistry, Faculty of Medicine, Istanbul Atlas University, Istanbul 34403, Türkiye; huzun59@hotmail.com; 3 Department of Analytical Chemistry, Faculty of Pharmacy, Istanbul University, Istanbul 34116, Türkiye; durisehvar@gmail.com; 4 Drug Research and Application Center, Istanbul University, Istanbul 34116, Türkiye

**Keywords:** cigarette smoke, inflammation, immunopathogenesis, oxidative stress, NLRP3 inflammasome, epigenetic reprogramming

## Abstract

Cigarette smoking is the leading preventable cause of chronic diseases (e.g., COPD, cardiovascular disease, cancer), largely driven by persistent immune-inflammatory mechanisms. This review synthesizes the molecular and cellular cascades linking cigarette smoke (CS) exposure to chronic pathology. CS constituents, particularly ROS/RNS, induce rapid oxidative stress that overwhelms antioxidant defenses and generates damage-associated molecular patterns (DAMPs). These DAMPs activate pattern recognition receptors (PRRs) and the NLRP3 inflammasome, initiating NF-κB signaling and the release of pro-inflammatory cytokines (TNF-α, IL-1β, IL-6). CS exposure causes profound innate immune dysregulation, including airway epithelial barrier disruption, hyperactivated neutrophils, and dysfunctional alveolar macrophages (AMs) that release destructive proteases (e.g., MMP-12) and acquire foam-cell–like characteristics. Furthermore, CS drives adaptive immunity toward a Th1/Th17-dominant phenotype while suppressing regulatory T-cell (Treg) function, thereby promoting autoimmunity and chronic tissue injury. Critically, CS induces epigenetic reprogramming (e.g., DNA methylation, miRNA dysregulation), locking immune cells into a persistent pro-inflammatory state. This convergence of oxidative stress, innate and adaptive immune dysregulation, and epigenetic alterations underlies the systemic low-grade inflammation that fuels smoking-related chronic diseases, highlighting key targets for novel therapeutic interventions.

## 1. Introduction

Cigarette smoking remains one of the leading preventable causes of morbidity and mortality worldwide. According to the World Health Organization (WHO), approximately 1.1 billion people smoke cigarettes globally, and tobacco use is responsible for more than 7 million deaths each year (WHO, 2021) [1]. This striking burden underscores the global impact of tobacco consumption as a modifiable risk factor for premature death and highlights the urgent need for both public-health interventions and mechanistic research aimed at mitigating its consequences.

The harmful effects of smoking are mediated through complex immune-inflammatory mechanisms that contribute to both local tissue injury and systemic pathology. Exposure to cigarette smoke (CS) initiates a multi-level cascade, beginning with oxidative stress and activation of the innate immune system, followed by dysregulation of adaptive immunity, and ultimately culminating in persistent systemic inflammation that underlies the pathogenesis of many chronic diseases. For example, CS-induced inflammation is central to the development of chronic obstructive pulmonary disease (COPD), atherosclerosis, metabolic disorders, infection susceptibility, and cancer [2,3,4].

Both active and passive inhalation of CS result in the rapid dissolution of toxic constituents into the epithelial lining fluid of the oral cavity and respiratory tract, followed by systemic absorption of reactive and harmful compounds [2]. Consequently, not only smokers but also individuals exposed to second-hand smoke experience widespread distribution of injurious agents, leading to epithelial injury, oxidative stress, and immune-inflammatory activation at mucosal surfaces and beyond.

In the respiratory tract, CS exposure damages airway epithelial cells, increases epithelial permeability, induces mucus hypersecretion, impairs mucociliary clearance, and drives the recruitment of macrophages and neutrophils, which release pro-inflammatory cytokines such as interleukin (IL)-6, IL-8, and tumor necrosis factor-α (TNF-α)⁠ [5,6]. Mechanistically, activation of pattern-recognition receptors (PRRs) such as Toll-like receptor 4 (TLR4) and IL-1 receptor 1 (IL-1R1), along with downstream MyD88 signaling, is required for acute CS-induced neutrophil recruitment and cytokine release in murine models. CS can also activate the NLRP3 inflammasome and related pyroptotic pathways in pulmonary and systemic immune cells. Although human studies have reported heterogeneous findings regarding the effects of CS on NLRP3 inflammasome activation, more recent evidence indicates that mitochondria-derived damage-associated molecular patterns (DAMPs) induced by CS can trigger NLRP3 activation, thereby exacerbating pulmonary inflammation and promoting atherosclerosis [3,4].

**Figure 1:**
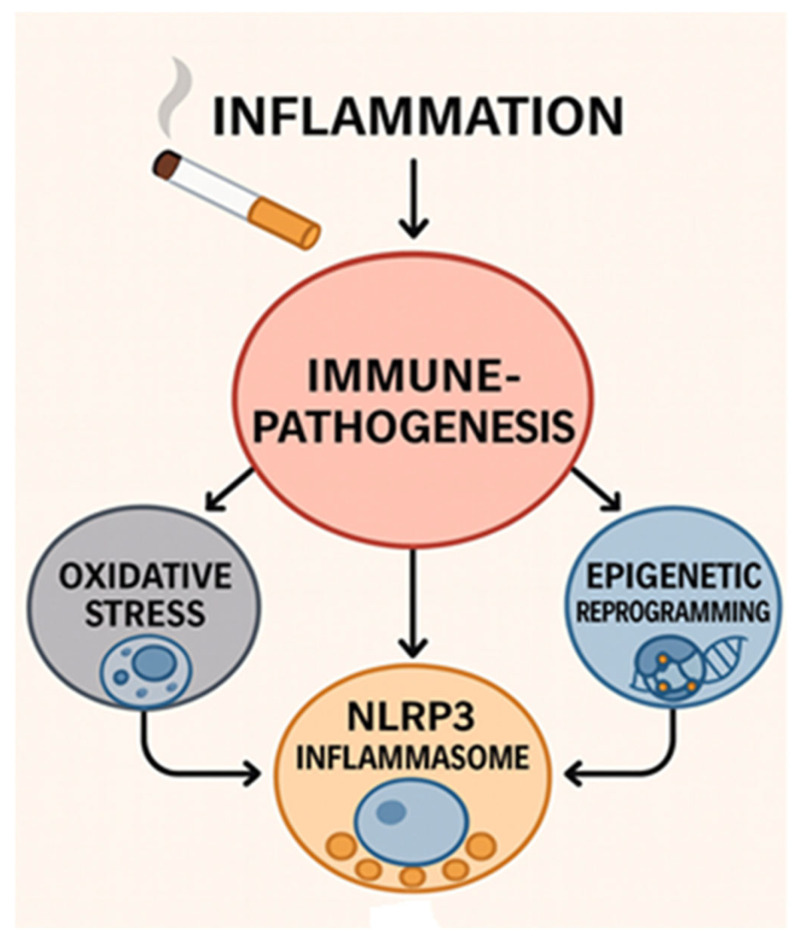
Convergent Pathways of Cigarette Smoke-Induced Immunopathogenesis.

Figure 1 summarizes the fundamental immune-inflammatory mechanisms by which CS exposure leads to chronic inflammation and disease pathogenesis. The oxidative stress caused by CS initiates immunopathogenesis by leading to cellular damage and the accumulation of ROS. This process can contribute to the activation of the inflammasome, which amplifies inflammatory signals and triggers the release of pro-inflammatory cytokines. Simultaneously, CS induces epigenetic reprogramming (including DNA methylation, histone modifications, and miRNA dysregulation), permanently altering the function of immune cells and sustaining a pro-inflammatory phenotype. These three key mechanisms (oxidative stress, activation of the inflammasome, and epigenetic reprogramming) collectively form the multifaceted immunopathogenesis that drives the development of tobacco-related chronic diseases.

Within the cardiovascular system, smoking-induced oxidative stress and immune activation contribute to endothelial dysfunction, upregulation of adhesion molecules (e.g., ICAM-1, VCAM-1), platelet activation, and a pro-thrombotic state, thereby promoting the initiation and progression of atherosclerotic and atherothrombotic disease⁠ [3,4,5,6,7]. At the same time, dysregulation of adaptive immunity—such as altered regulatory T-cell (Treg) responses, impaired complement C1q signaling, and skewing toward pro-inflammatory Th17 phenotypes—has been documented in smokers and may contribute to loss of peripheral immune tolerance and increased disease susceptibility⁠ [8].

Given the multiplicity and interconnectedness of these immune-inflammatory pathways, a comprehensive understanding of how CS induces, sustains, and propagates inflammation is critical. This literature review, therefore, aims to examine in depth the immune and inflammatory mechanisms triggered by CS and to discuss how these mechanisms contribute to the development of chronic diseases, including COPD, cardiovascular disease and atherosclerosis, metabolic disorders, infection susceptibility, and cancer⁠ [9,10,11,12,13]. This paper constitutes a narrative review of the current literature on the mechanisms of smoke-induced immunopathogenesis.

In contrast to previous review articles that mainly examined individual pathways or disease-specific outcomes of CS exposure, this review presents an integrative framework of convergent immunopathogenesis. By combining oxidative stress, innate and adaptive immune dysregulation, and epigenetic reprogramming into a unified mechanistic continuum, it emphasizes how CS triggers a persistent, system-wide inflammatory memory that crosses organ boundaries. This systems-level approach offers a conceptual link between molecular mechanisms and multi-organ disease symptoms, providing a translational framework to identify shared therapeutic targets and guide public health efforts aimed at preventing smoking-related chronic diseases.

## 2. Hazardous Compounds in Cigarette Smoke

Importantly, CS contains more than 7000 chemicals, many of which exhibit cytotoxic, mutagenic, carcinogenic, or antigenic properties, further amplifying its pathogenic potential [5].

These compounds belong to multiple chemical classes, including volatile organic compounds, alkaloids, aldehydes, polycyclic aromatic hydrocarbons (PAHs), nitrosamines, heavy metals, and free radicals. The gas phase of CS contains carbon monoxide, nitrogen oxides, formaldehyde, acetaldehyde, acrolein, and other reactive species, whereas the particulate (tar) phase concentrates PAHs, nicotine, and high-molecular-weight mutagens such as benzo[a]pyrene [14].

**Figure 2:**
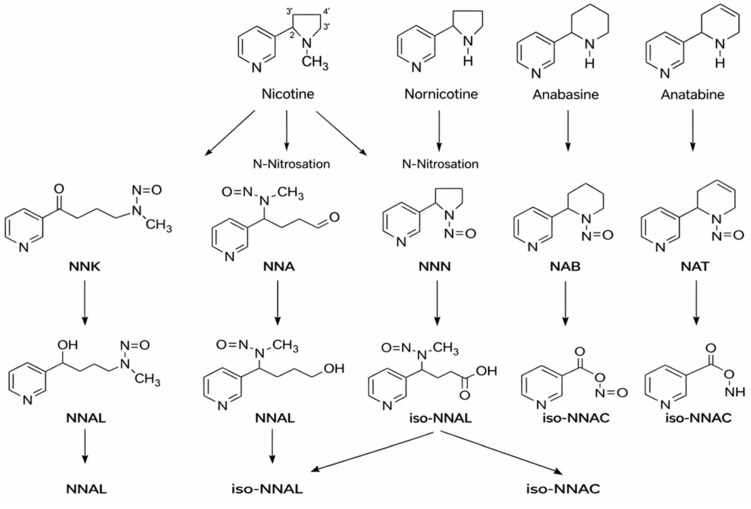
Tobacco alkaloids and formation of related tobacco-specific nitrosamines [16].

The main alkaloid found in tobacco is nicotine, and the most known and abundant minor tobacco alkaloids are nornicotine, anatabine, and anabasine. All of these alkaloids are amines (Figure 2). The nitrite found in saliva, and the nitrogen oxides present in inhaled mainstream of tobacco smoke, reacted with the alkaloids which contain an amine group in their structure, and formed tobacco-specific nitrosamines (TSNAs) [15].

A particularly well-studied subgroup is the tobacco-specific nitrosamines, which arise from the nitridation of nicotine and structurally related alkaloids and represent potent, organ-specific carcinogens in experimental models. The best-known TSNAs include: (1) 4-(methylnitrosamino)-1-(3-pyridyl)-1-butanone (NNK) and its metabolite 4-(methylnitrosamino)-1-(3-pyridyl)-1-butanol (NNAL), both formed through the nitrosation of nicotine; (2) N′-nitrosonornicotine (NNN), derived from the nitrosation of nornicotine; (3) N′-nitrosoanabasine (NAB), formed from anabasine; and (4) N′-nitrosoanatabine (NAT), generated from anatabine (Table 1). NNK and NNN can be found in all brands of tobacco. Nitrosamines are absorbed through the alveoli and airways of the lung and distributed by the blood to the body. NNN can be endogenously formed from nicotine and nornicotine. To evaluate the potential health risk of cigarette smoking or exposure can be evaluated by using TSNAs as biomarkers [16].

These TSNAs are among the most potent carcinogenic constituents of tobacco smoke, capable of inducing DNA adduct formation, mutations, and tumorigenesis in multiple organ systems, including the lung, esophagus, and pancreas. Their formation occurs during tobacco curing, processing, and combustion, and their metabolic activation by cytochrome P450 enzymes produces reactive intermediates that covalently bind to DNA and proteins, initiating mutagenic and inflammatory cascades that underlie tobacco-related carcinogenesis [17,18].

## 3. Oxidative Stress and Activation of Inflammatory Pathways

CS is a complex mixture containing thousands of toxic chemicals, including abundant reactive oxygen and nitrogen species (ROS/RNS) that impose severe oxidative stress on biological systems. Upon inhalation, these reactive species directly interact with airway epithelial cells, alveolar macrophages, and endothelial cells, initiating a cascade of oxidative and inflammatory responses. The excessive production of ROS and RNS overwhelms the endogenous antioxidant defense mechanisms such as superoxide dismutase (SOD), catalase, and glutathione peroxidase, resulting in lipid peroxidation, protein oxidation, and DNA damage [19].

The oxidative modification of membrane lipids destabilizes cellular membranes, alters permeability, and disrupts ion gradients, thereby impairing cellular homeostasis and promoting necrotic or apoptotic cell death. Lipid peroxidation products, such as malondialdehyde (MDA) and 4-hydroxynonenal (4-HNE), act as secondary messengers that perpetuate oxidative injury by forming covalent adducts with proteins and nucleic acids, amplifying cellular dysfunction and inflammatory signaling [19,20,21].

Protein oxidation leads to structural denaturation, enzyme inactivation, and loss of receptor activity, thereby disturbing crucial cell signaling, energy metabolism, and immune regulation. Simultaneously, oxidative DNA damage results in strand breaks, base modifications, and chromosomal aberrations, promoting mutagenesis, genomic instability, and the activation of cell death pathways such as apoptosis and necroptosis. These cumulative changes weaken the epithelial and endothelial barriers, increase vascular permeability, and trigger tissue remodeling, hallmark processes in chronic lung injury [20,21].

The cellular stressors derived from oxidative damage, including lipid peroxidation products and DAMPs, engage PRRs such as the receptor for advanced glycation end products (RAGE) and Toll-like receptors (TLRs). Activation of these receptors stimulates pro-inflammatory signaling cascades, notably through the mitogen-activated protein kinase (MAPK) and nuclear factor-kappa B (NF-κB) pathways, leading to the upregulation of cytokines (e.g., TNF-α, IL-6, IL-8) and adhesion molecules, which recruit neutrophils and macrophages to the site of injury [19,20,21,22].

In chronic or heavy smokers, sustained oxidative stress is evidenced by elevated circulating and tissue levels of MDA, nitric oxide (NO), and endothelin-1, along with diminished antioxidant capacity [23]. These biochemical disturbances are accompanied by endothelial dysfunction, impaired nitric oxide bioavailability, and vascular inflammation, creating a pro-atherogenic and pro-fibrotic microenvironment. Consequently, persistent oxidative and inflammatory insults contribute not only to chronic obstructive pulmonary disease (COPD), acute respiratory distress syndrome (ARDS), lung cancer, and pulmonary fibrosis, but also to systemic effects such as cardiovascular diseases, metabolic syndrome, and accelerated aging.

In the study of Ahmadkhaniha et al., total antioxidant capacity (TAC), total oxidant status (TOS), and oxidative stress index (OSI) levels were evaluated in smokers, passive smokers, and nonsmokers. TAC levels were significantly greater in nonsmokers than in smokers and passive smokers, whereas TOS and OSI levels were significantly higher in smokers [24]. In another study conducted in mice, Short-term inhalation exposure to CS led to an increase in neutrophils, eosinophils, and total cell counts in bronchoalveolar lavage fluid. It also elevated levels of lactate dehydrogenase and MDA, indicative of tissue damage and oxidative stress, respectively [25]. Another study demonstrated that CS exposure to cultured cerebral vascular smooth muscle cells increased expression of NADPH oxidase (NOX) and ROS, which preceded upregulation of proinflammatory/matrix remodeling genes (Monocyte Chemoattractant Protein-1 (MCP-1), matrix metalloproteinase (MMP), TNF-α, IL-1β, NF-κB, Kruppel-like factor 4 (KLF-4)) [26].

## 4. Innate Immune Dysregulation

### 4.1. Airway Epithelial and Alveolar Responses

The airway epithelium is the first interface between inhaled smoke and the lung. Damage here sets off cascades of inflammation, remodeling, and eventual disease (e.g., COPD, emphysema, lung cancer). Downstream, smoke reaches alveoli, where alveolar epithelial cells, surfactant-producing cells, and alveolar macrophages respond and can become dysfunctional. CS disrupts tight junctions (TJs) and adherent junctions in airway epithelium, compromising barrier integrity. CS also induces epidermal growth factor receptor (EGFR) dependent signaling that contributes to junctional disruption and “leaky” epithelium. This disruption increases epithelial permeability and can allow deeper penetration of toxins, allergens, and pathogens [27]. There is also evidence for epithelial–mesenchymal transition (EMT) induced by chronic smoke exposure, contributing to structural remodeling of the airway wall [28]. EMT is a process involved in cell migration, repair, and tissue remodeling, with loss of epithelial markers and junctional proteins and gain of mesenchymal markers. These epithelial changes induce expression of growth factors, MMP levels, and extracellular matrix, leading to subepithelial fibrosis, an important hallmark of COPD.

CS has also been found to affect key genes involved in lung senescence, such as fibronectin, MMPs, and sirtuin-1 (SIRT1). These effects lead to decreased fibronectin levels and increased MMP levels, which contribute to the loss of lung function and the development of respiratory diseases. Downregulation of SIRT1 in lung epithelium disrupts its protective role against lung injury and senescence [20].

CS leads to ciliary dysfunction, shortened cilia, and reduced ciliary beat frequency, thereby impairing mucociliary clearance. There is also a shift from normal epithelial cell types toward more mucus-secreting (goblet) cells (goblet cell hyperplasia/metaplasia), increasing mucus production and potentially leading to mucus accumulation. Over time, this contributes to chronic bronchitis features (excess mucus, airway obstruction) [29]. Basal/progenitor (stem) cells are also affected: smoke may skew differentiation trajectories, reduce regenerative capacity, or cause aberrant repair (favoring secretory cells over ciliated, etc.) [28].

Alveolar epithelial type I and type II cells can be directly injured by reactive species, leading to mitochondrial damage, oxidative stress, and cell death. In particular, in vitro studies show that mild exposures to CS impair mitochondrial function (e.g., reducing mitochondrial membrane potential, increasing ROS) in alveolar epithelial lines [30]. Chronic injury triggers remodeling of alveolar septa: loss of alveolar walls, enlargement of airspaces (emphysematous changes), destruction of capillary beds, and loss of alveolar surface area. CS can also interfere with surfactant production and composition by alveolar type II cells, reducing the effectiveness of the surfactant layer. Disrupted surfactant function increases surface tension, predisposes to alveolar collapse (atelectasis), and impairs gas exchange [31].

Alveolar macrophages (AMs) play a critical role in maintaining surfactant homeostasis and alveolar stability. Under physiological conditions, AMs regulate the surfactant layer that coats the alveolar epithelium by phagocytosing and degrading damaged or oxidized surfactant components produced by alveolar epithelial type II cells. This clearance process ensures optimal surfactant composition and function, thereby maintaining low alveolar surface tension and preventing atelectasis [32]. Approximately 50% of surfactant turnover is mediated by AMs through enzymatic degradation and lipid recycling, while the remaining portion is managed by AT2 cells via endocytic uptake, recycling, and de novo synthesis of surfactant phospholipids and proteins [33]. The development, differentiation, and functional maturation of AMs depend on granulocyte–macrophage colony-stimulating factor (GM-CSF) signaling, primarily through CSF2 receptor (CSF2RA/CSF2RB). Experimental studies in GM-CSF-deficient mice demonstrate impaired AM development, leading to the accumulation of surfactant and lipoproteinaceous material within the alveoli—a phenotype resembling pulmonary alveolar proteinosis (PAP) [34]. Following CS exposure, IL-1α released from injured epithelial cells stimulates GM-CSF production. Sustained pulmonary GM-CSF overexpression promotes macrophage accumulation, heightened MMP-12 secretion, and matrix degradation, leading to diffuse interstitial pneumonia and emphysematous remodeling—characteristic features of smoking-associated parenchymal lung disease. In addition, AMs exposed to CS exhibit dysfunctional surfactant processing, characterized by excessive uptake and incomplete degradation of oxidized lipids, resulting in lipid accumulation and foam cell formation—a hallmark of smoke-induced macrophage pathology in AMs (a defining feature of foam cells) [33]. AMs in smokers display altered recruitment, phenotypes, and impaired phagocytic function, contributing to ineffective pathogen clearance and persistent inflammation [35].

### 4.2. Neutrophil Activation

Exposure to CS activates airway epithelial cells to release TNF-α, IL-1β, IL-6, IL-8, and leukotriene B4, and generates chemotactic factors recruiting neutrophils and macrophages into the airways [33,36]. CS exposure activates the pro-inflammatory transcription factor NF-κB and increases messenger RNA (mRNA) expression of IL-8 (one of the mediators controlled by NF-κB) within the macrophages [37]. In an ex vivo study, whole-blood samples from smokers, when incubated without any exogenous stimulus, secreted significantly higher levels of the neutrophil chemoattractant IL-8 compared with samples from non-smoking controls. A similar, though less pronounced, trend toward elevated cytokine and chemokine release was observed for multiple mediators, including IL-1β, granulocyte colony-stimulating factor (G-CSF), IL-13, IL-17, vascular endothelial growth factor (VEGF), interferon-γ (IFN-γ), IL-12, interferon-α (IFN-α), IL-4, and macrophage inflammatory protein-1α (MIP-1α) after 7 h incubation of unstimulated blood samples of smoker [35]. When neutrophils become hyperactivated, they produce ROS and release extracellular traps and destructive proteases (e.g., MMPs), exacerbating tissue damage and amplifying inflammation [33].

### 4.3. Impairment of Other Innate Cells

Chronic smoke exposure impairs dendritic cell maturation and IFN-α production, and suppresses NK cell cytotoxic functions, including reduced IFN-γ and TNF-α expression, thus increasing vulnerability to infections [35].

## 5. Adaptive Immune Responses and Epigenetic Alterations

Smoking disrupts the balance between effector and regulatory T-cells, enhancing Th1 and Th17 responses while suppressing Tregs, potentially promoting autoimmunity and chronic tissue injury [38]. CS exposure promotes a shift in adaptive immunity characterized by T helper 1 (Th1) and T helper 17 (Th17) polarization. This results in increased production of signature cytokines such as interferon-γ (IFN-γ), interleukin-12 (IL-12), and interleukin-17 (IL-17) [39]. IL-12 released from antigen-presenting cells (macrophages and dendritic cells) drives Th1 differentiation, leading to increased IFN-γ secretion. IFN-γ further enhances macrophage activation and the production of ROS/RNS, amplifying tissue injury and chronic inflammation in the airway and alveoli. CS and its oxidants induce dendritic cell maturation and promote IL-6 and TGF-β production, favoring Th17 differentiation [40]. This Th1/Th17-dominant immune milieu promotes persistent neutrophilic inflammation, MMP activation, and alveolar destruction while impairing resolution pathways mediated by Th2 and regulatory T cells (Tregs) [41].

CS also induces epigenetic changes, including DNA methylation alterations, histone modifications (e.g., increased acetylation of H3K9, H4K12), and dysregulation of miRNAs (e.g., downregulation of miR-146a) that modulate immune gene expression and contribute to chronic inflammation and carcinogenesis [42].

## 6. Systemic Immune Dysregulation and Disease Manifestations

Smoking exerts widespread and deleterious effects on virtually every major organ system in the human body. Local pulmonary inflammation induced by CS triggers a persistent, low-grade systemic inflammatory state, as evidenced by elevated white blood cell count, C-reactive protein (CRP), fibrinogen, and pro-inflammatory cytokines such as IL-1β and IL-6 [28]. Beyond its well-established respiratory consequences, smoking is now recognized as a primary causal driver of numerous chronic and systemic diseases. Elevated levels of CRP, fibrinogen, and leukocytes are consistently associated with an increased risk of multiple cancer types as well as cardiovascular disease. Notably, pharmacological targeting of IL-1β with canakinumab has been shown to reduce lung cancer incidence among smokers, providing compelling evidence for the central role of systemic inflammation in smoking-related carcinogenesis [35].

Importantly, while oxidative stress and inflammation represent common upstream processes, the downstream immune pathways activated by CS differ substantially across organ systems. In the pulmonary compartment, the release of DAMPs and engagement of PRRs predominantly drive neutrophil and macrophage activation, leading to protease-mediated tissue destruction and chronic airway remodeling. In contrast, within the cardiovascular system, CS-induced immune activation preferentially targets endothelial cells, promoting endothelial dysfunction, leukocyte adhesion, platelet activation, and foam cell formation—key processes in atherogenesis [33,35,38,42]. In metabolic and renal tissues, CS-mediated reprogramming of immunometabolic pathways in innate immune cells and T lymphocytes amplifies low-grade inflammation, insulin resistance, and fibrotic remodeling, whereas in the central nervous system, similar inflammatory triggers interact with microglial activation and immune-privileged barriers to promote neurodegenerative processes.

Across these diverse tissues, CS consistently activates NF-κB signaling through PRRs pathways, leading to the release of cytokines, chemokines, matrix metalloproteinases (MMPs), and additional DAMPs. However, the biological consequences of these pathways are shaped by the local cellular and metabolic context of each organ system. Together, these shared yet context-dependent mechanisms illustrate that smoking-induced oxidative stress does not produce a uniform inflammatory response, but rather drives distinct immune phenotypes that converge on chronic inflammation, tissue injury, and long-term disease progression [30,42].

Epigenetic reprogramming represents a critical mechanistic bridge between acute immune activation and the persistence of chronic inflammation. CS induced oxidative stress and innate immune signaling initiate transcriptional programs that are subsequently stabilized through epigenetic modifications, including DNA methylation changes, histone remodeling, and microRNA dysregulation [42]. These alterations imprint immune cells with a form of immune memory–like state, leading to exaggerated or dysregulated responses upon repeated or prolonged exposure. As a result, innate and adaptive immune cells remain locked in a pro-inflammatory phenotype, even in the absence of continuous stimulation, thereby linking early immune activation to long-term systemic immune dysregulation and disease progression. Recent evidence from large-scale human epigenomic analyses demonstrates that cigarette smoking induces long-lasting epigenetic reprogramming of immune cells, with DNA methylation changes at loci such as AHRR, F2RL3, and GPR15 accounting for a significant proportion of inter-individual variability in cytokine responses, persisting up to 10–15 years after smoking cessation [43].

**Figure 3:**
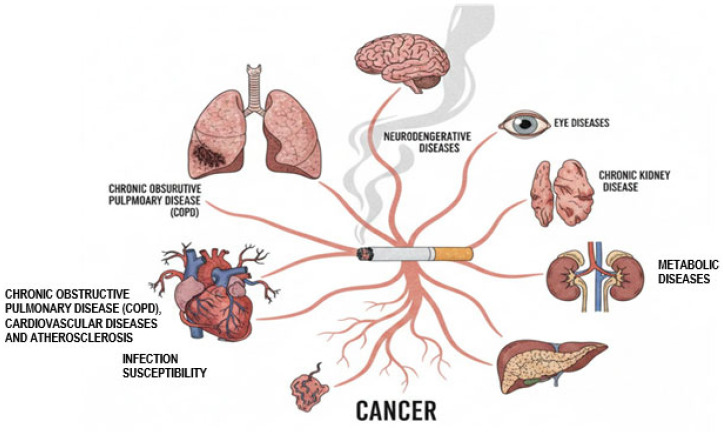
Smoking-Induced Systemic Pathologies: A Multi-Organ Perspective.

As illustrated in Figure 3, the toxic compounds in CS are absorbed into the bloodstream, initiating damage throughout the body. This mechanism directly links smoking to the development or exacerbation of several serious conditions:

**Pulmonary System (Lungs):** A major contributor to the pathogenesis of chronic obstructive pulmonary disease (COPD).

**Cardiovascular System (Heart and Vessels):** Causes endothelial injury leading to cardiovascular disease and atherosclerosis, while impairing immune defenses and increasing susceptibility to infections.

**Central Nervous System (Brain and Nerves):** Significantly contributes to the risk and progression of various neurodegenerative diseases.

**Renal and Metabolic Systems (Kidneys and Metabolism):** Strongly associated with the development of chronic kidney disease and multiple metabolic disorders, including type 2 diabetes.

**Ophthalmic System (Eyes):** Increases the incidence and severity of several eye diseases, such as cataracts and age-related macular degeneration.

**Systemic Malignancy (Multiple Organs):** Substantially elevates the lifetime risk of various cancers affecting multiple organ systems.

### 6.1. Chronic Obstructive Pulmonary Disease (COPD)

Smoking is the most common and significant cause of chronic obstructive pulmonary disease (COPD), with approximately 80% of COPD patients having a history of cigarette smoking. Continuous exposure to inhaled irritants represents the most critical etiological factor in COPD, and the prominent role of smoking in this process has been strongly emphasized in numerous studies [44,45,46].

Our findings have substantial implications for both policy development and future research. Smoking remains the predominant modifiable risk factor for COPD, underscoring the need for detailed assessment of smoking behaviors across age groups and the adoption of rigorous tobacco-control measures. Although the epidemiological understanding of COPD remains limited in many LMICs, broad and effective implementation of the WHO Framework Convention on Tobacco Control—ratified by 180 parties representing 90% of the global population—will be essential for reducing the global COPD burden [47]. Priority should be given to low- and middle-income countries, where disease prevalence is highest, regulatory measures against the expanding tobacco industry are insufficient, and smoking rates among young people continue to rise. Strengthening tobacco-dependence treatment efforts led by physicians, other health professionals, and structured counseling and support systems is critical, especially because evidence-based interventions are particularly effective among individuals who are motivated to quit [48]. Given the limitations of available population-based datasets, experts should work toward harmonized approaches for collecting clinically relevant data that can support epidemiological analyses. As a global respiratory health body, we advocate for routinely documenting pack-years during the clinical evaluation of patients presenting with breathlessness or chronic cough. Addressing COPD misdiagnosis is also imperative—particularly by improving access to spirometry and enhancing provider training in its appropriate use. This need is especially pronounced in sub-Saharan Africa and South-East Asia, where undiagnosed COPD remains highly prevalent [49]. In addition, improved training and clear clinical guidance are required to optimize and individualize pulmonary rehabilitation, which has demonstrated proven efficacy in COPD management [50].

Smoke-induced inflammation and oxidative damage degrade alveolar walls, impair antiprotease activity (e.g., α1-antitrypsin), and promote airway remodeling, driving emphysema and chronic bronchitis pathogenesis [20,21].

Recent single-cell and spatial transcriptomics studies have provided critical mechanistic insights into the pathogenic continuum linking epithelial dysfunction to chronic inflammation and downstream metabolic and epigenetic reprogramming in COPD. In a large single-cell RNA sequencing analysis of 111,540 lung cells, Sauler et al. identified a disease-specific AT2B alveolar epithelial subpopulation characterized by aberrant metabolic programming, impaired stress tolerance, and disrupted cellular homeostasis, features that likely lower the threshold for CS induced epithelial injury [51]. The same study also described high–metallothionein–expressing macrophages uniquely enriched in COPD lungs, reflecting adaptive responses to sustained oxidative and metal stress but concurrently exhibiting transcriptional profiles consistent with altered inflammatory set points [51].

These epithelial and immune cell alterations provide a plausible cellular basis for persistent epithelial–immune crosstalk, promoting the chronic release of ROS, DAMPs, and alarmins that perpetuate innate immune activation. Importantly, a recent large-scale integrative atlas combining single-cell and spatial transcriptomics across approximately 9.2 million cells further demonstrated that these maladaptive epithelial and immune cell states are spatially organized within inflamed and structurally remodeled lung niches [52]. This spatial persistence supports a feed-forward model in which epithelial dysfunction initiates sustained inflammation, which in turn stabilizes disease-specific metabolic and epigenetic programs resembling trained immunity, thereby contributing to the chronicity and partial irreversibility of COPD-related inflammation even after smoking cessation [52].

### 6.2. Cardiovascular Diseases and Atherosclerosis

Persistent airway inflammation and heightened oxidative stress induced by CS exposure are believed to play a central role in the development of atherosclerosis and cardiovascular disease among individuals with COPD. Growing evidence indicates that pro-inflammatory mediators and ROS can enter the systemic circulation, thereby contributing to pathological processes beyond the lungs. Previous research demonstrates that CS triggers not only pulmonary inflammation but also systemic inflammatory responses, oxidative stress, endothelial dysfunction, and increased circulating pro-coagulant factors. These widespread systemic effects of CS promote the development of chronic comorbid conditions and further functional decline, ultimately diminishing patients’ overall quality of life [53].

A review by Ishida et al. [3] examines the fundamental mechanisms of cigarette smoking in the development of atherosclerotic cardiovascular disease (CVD) under three main headings: endothelial dysfunction, inflammation, and thrombotic processes [2]. Toxic compounds and ROS in CS impair vasodilation by reducing nitric oxide bioavailability, increasing endothelin release, and leading to significant endothelial damage. This process, combined with mitochondrial dysfunction and increased oxidative stress, triggers the early stages of atherosclerosis. Smoking also induces a strong systemic inflammatory response. Key immune pathways such as the NLRP3 inflammasome, cGAS–STING, and TLR9 are activated, along with an increase in adhesion molecules (ICAM-1, VCAM-1) and pro-inflammatory cytokines. These mechanisms perpetuate chronic inflammation in the vascular wall, accelerating atherosclerotic plaque development. Thrombotic effects also significantly increase the risk of CVD. Smoking enhances platelet activation, aggregation, and the coagulation cascade, while reducing fibrinolytic activity. Increased levels of matrix metalloproteinases, in turn, predispose to plaque instability, increasing the risk of acute cardiovascular events. The review emphasizes that smoking cessation is the most effective approach to reducing CVD risk and notes that new treatments targeting inflammation and thrombosis hold significant potential in the future [2].

Smoking impairs endothelial function (by reducing nitric oxide and inducing endothelin-1), fosters oxidized LDL uptake by macrophages to form foam cells, increases platelet adhesion, and promotes a prothrombotic environment [54].

### 6.3. Infection Susceptibility

A study by Kjerulff and colleagues, published in 2023, demonstrated that smoking significantly increases the risk of infection through a large-scale analysis of healthy blood donors [55]. Because the donors were generally healthy individuals, the study was able to assess the impact of smoking on infection susceptibility independent of other diseases. The researchers found higher rates of both respiratory and systemic infections among smokers, as well as increased antibiotic use. These findings are consistent with the well-established effects of smoking on impairing mucosal defenses, weakening immune cell function, and disrupting the balance between inflammation and oxidative stress. The observation that infection risk rises with increasing amounts of smoking further supports the dose-dependent nature of this relationship. Overall, the study emphasizes that smoking poses a major public health risk not only for chronic diseases but also for acute infections, highlighting the critical importance of smoking-cessation programs in reducing infection-related health burdens [55].

Compromised innate immunity, impaired phagocytosis, defective dendritic and NK cells render smokers more susceptible to respiratory infections such as pneumonia and tuberculosis [24].

**Figure 4:**
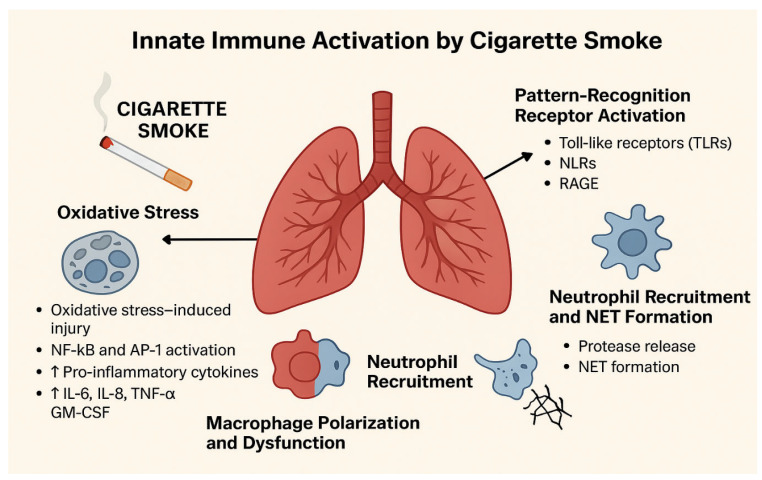
Cigarette Smoke–Induced Activation of Innate Immunity in the Lungs.

CS represses several well-characterized components of the innate immune response to inhaled asbestos [56]. Figure 4 illustrates how CS triggers the innate immune response in the lungs. CS initially causes cellular damage through oxidative stress and activation of NF-κB and AP-1, which subsequently leads to increased production of pro-inflammatory cytokines such as IL-6, IL-8, TNF-α, and GM-CSF. This is followed by macrophage dysfunction and polarization, recruitment of neutrophils to the lungs, and the formation of neutrophil extracellular traps (NETs) [57]. Furthermore, the immune system’s inflammatory response is amplified through the activation of PRRs, including TLRs, NLRs, and RAGE. These processes contribute to the development of COPD, cardiovascular disease, and other smoking-related pathologies by promoting chronic inflammation and tissue damage [58].

### 6.4. Cancer

Cigarettes and CS contribute to the development of many types of cancer, particularly lung cancer. Some of these cancers arise in organs directly exposed to CS, such as the respiratory tract and oral cavity, while others develop in organs that are not directly exposed. Tar in tobacco smoke is one of the most important carcinogenic components involved in cancer development. In addition, among the thousands of chemicals released during tobacco combustion, many are known carcinogens. These substances enter the bloodstream, disseminate throughout the body, and can induce cancer in multiple organs. According to current evidence, tobacco use directly contributes to the development of at least ten different types of cancer [59,60,61,62,63,64,65,66].

While 90% of lung cancers are caused by smoking, smoking also plays a significant role in the development of cancers of the larynx, oral cavity, pharynx, esophagus, stomach, colon, pancreas, kidney, bladder, breast, and cervix. Cigarettes are responsible for approximately one-third of all human cancers [59,60,61,62,63,64,65,66]. Chronic inflammation, oxidative DNA damage, and compromised immune surveillance foster carcinogenesis, especially in the lungs and vascular tissues [35,42].

A review by Kim, Park, and Chiang published in JAMA clearly demonstrates the impact of smoking on small-cell lung cancer (SCLC). SCLC is a tumor type that is strongly (~95%) associated with smoking, highlighting smoking as the most significant risk factor for this aggressive malignancy. Although current treatment strategies combine immunotherapy with traditional chemotherapy and radiotherapy, overall survival remains limited due to challenges in early diagnosis and the high propensity for recurrence. This underscores the critical importance of smoking cessation in preventive medicine. Furthermore, the review offers a promising outlook on the potential to therapeutically target the genetic and cellular damage caused by smoking, focusing on emerging approaches directed at key biological features of SCLC (e.g., DLL3-targeted agents) [60].

### 6.5. Metabolic Diseases

Metabolic diseases such as diabetes mellitus (DM) [67], metabolic dysfunction–associated fatty liver disease (MAFLD) [68], hypercholesterolemia [69], and obesity [70] constitute a rapidly increasing public health burden worldwide. This growing burden affects not only individual health but also economic sustainability [71]. While a strong association between cigarette smoking and metabolic diseases has been clearly documented, the mechanistic basis underlying this relationship has not yet been fully elucidated [72,73,74,75]. Current evidence suggests that smoking predisposes individuals to metabolic dysfunction by impairing insulin sensitivity, amplifying inflammatory responses, and increasing oxidative stress [76,77,78,79,80]. However, more comprehensive research is needed to fully elucidate the multifaceted interactions among these biological processes [80].

The association between cigarette smoking and metabolic dysfunction is well established, with compelling evidence demonstrating its role in increasing susceptibility to impaired glucose tolerance and accelerating the incidence of diabetes in the general population [81]. This comprehensive review and joint statement by Durlach and colleagues systematically examine the mechanisms and clinical consequences of the complex relationship between CS and diabetes mellitus (DM), addressing both Type 1 (T1DM) and Type 2 (T2DM). The authors conclude with a strong clinical recommendation emphasizing the critical importance of smoking cessation: quitting smoking provides rapid and substantial benefits, improving insulin sensitivity, enhancing glycemic control, and significantly reducing both microvascular and macrovascular risks in patients with diabetes. The review advocates for aggressive, multidisciplinary strategies to promote and support smoking cessation among all individuals with DM [81].

A modeling study by Sözmen and colleagues demonstrates that the projected 2025 diabetes prevalence in Turkiye is strongly influenced by trends in obesity and smoking [82]. The study suggests that if current patterns persist, the national burden of diabetes will rise substantially; however, effective interventions aimed at reducing obesity and smoking prevalence could lead to a meaningful decrease in diabetes rates. These findings highlight that community-level preventive strategies—particularly anti-obesity initiatives and tobacco control policies—are essential for altering the trajectory of the diabetes epidemic [82].

A review by Feldman and Anderson examines the major factors that increase the risk of community-acquired pneumonia (CAP) [83]. CS, alcohol consumption, diabetes mellitus, and metabolic syndrome have been identified as significant and independent risk factors for the development of CAP. Smoking increases susceptibility to infection by impairing mucociliary function in the airways and weakening immune defenses. Alcohol contributes to both immunosuppression and an increased risk of aspiration. Diabetes and metabolic syndrome elevate pneumonia risk through chronic inflammation and impaired glucose metabolism. The review emphasizes that the combination of these factors can substantially increase the incidence of CAP and highlights the importance of preventive strategies—such as smoking cessation, reducing alcohol consumption, and optimizing metabolic control—for protecting public health [83].

### 6.6. Chronic Kidney Disease

Although the detrimental impact of smoking on various organ systems is well established, its direct causal contribution to chronic kidney disease (CKD) has not been clearly defined. Beyond these well-known associations, smoking also poses significant risks to kidney health. Earlier studies have suggested potential harmful effects of smoking on renal function [84]. More recently, Yang et al. [85] conducted a two-sample Mendelian randomization study demonstrating that smoking has a causal effect on the development of CKD. Using genetically predicted smoking behaviors, the authors showed that smoking contributes to renal dysfunction independent of traditional confounders. Their findings provide robust genetic evidence supporting smoking as a modifiable risk factor for CKD and underscore the importance of tobacco control as a key component of kidney disease prevention strategies [85].

Smoking has been linked to impaired kidney health, yet evidence from previous research remains mixed. While some studies report that smoking reduces glomerular filtration rate (GFR) independently of proteinuria, others find no clear association with kidney function decline [86,87]. Additional reports describe associations between smoking, albuminuria, and abnormal renal function, although these effects appear weaker or absent in former smokers [88]. Experimental data suggest several potential mechanisms—including oxidative stress [89], inflammation [90], and activation of the renin–angiotensin–aldosterone system [91,92], which may promote vascular injury [93], and renal fibrosis [89,90,91,92,93]. However, these pathways are not fully elucidated and require further investigation.

### 6.7. Neurodegenerative Diseases

Neurodegenerative diseases encompass a spectrum of chronic, progressively worsening disorders of the nervous system, including Alzheimer’s disease (AD), Parkinson’s disease (PD), amyotrophic lateral sclerosis (ALS), multiple sclerosis (MS), and Lewy body dementia (LBD). These disorders are characterized by the gradual and selective degeneration of neurons in specific brain regions, leading to progressive structural and functional decline and resulting in cognitive, motor, or neuropsychiatric impairments [94]. Tobacco smoke contains an exceptionally diverse array of more than 9500 chemical constituents, 79 of which are confirmed carcinogens, while many others exert substantial toxic effects on human health [95]. Exposure to CS activates multiple harmful biological pathways, including inflammatory cascades, DNA damage, and aberrant methylation patterns, elevated oxidative stress, and a range of epigenetic alterations [7].

Chen et al. (2025) conducted a genome-wide analysis using both genetic correlation and Mendelian randomization approaches to investigate how smoking behaviors may causally influence neurodegenerative diseases [96]. They found that smoking initiation and higher cigarette consumption per day are genetically and causally associated with an increased risk of AD, whereas smoking cessation appears to reduce the risk of PD and ALS. These findings provide novel genetic evidence for the role of distinct smoking phenotypes in the pathogenesis of neurodegenerative disorders and suggest that smoking may contribute to AD risk through specific biological mechanisms, while quitting may confer protective effects against PD and ALS. Future studies should aim to clarify the precise biological pathways linking smoking-related behaviors to disease development, thereby supporting the design of more targeted and effective intervention strategies.

### 6.8. Eye Diseases

Smoking represents a substantial risk factor for a range of eye diseases due to multiple harmful mechanisms. Nicotine and other toxic constituents of tobacco impair microcirculation and promote oxidative stress and inflammatory processes. Growing evidence demonstrates that cigarette smoking is a major modifiable risk factor for a broad spectrum of ocular disorders, exerting detrimental effects through diverse inflammatory, vascular, and oxidative pathways. Barth et al. report that smoking significantly increases the risk and severity of several eye diseases—particularly age-related macular degeneration, cataract, uveitis, and retinal vascular pathology—by promoting oxidative stress, chronic inflammation, and microvascular injury [97]. Complementing these findings, Liu et al. provide mechanistic insights showing that smoking accelerates the progression of thyroid eye disease through activation of the RAGE signaling pathway, which enhances orbital inflammation, fibroblast activation, and tissue remodeling [98]. Collectively, these studies demonstrate that smoking not only contributes to the onset of multiple ocular disorders but also exacerbates disease progression and reduces therapeutic effectiveness. Understanding these shared pathogenic mechanisms underscores the necessity of smoking cessation as a central strategy for preventing vision impairment and reducing disease burden across ophthalmic conditions.

Cigarette smoking worsens neovascular age-related macular degeneration (nAMD) by activating pericytes through the Sema4D–PlexinB1 signaling pathway. Findings show that smoking-related stimuli increase Sema4D expression and downstream signaling, which promotes abnormal blood vessel growth and destabilizes the retinal microvascular environment. These mechanistic insights provide strong experimental evidence that smoking directly speeds up nAMD progression through pericyte-mediated vascular problems, highlighting the Sema4D–PlexinB1 pathway as a potential therapeutic target for smoking-related retinal disease [99].

Smoking may increase the risk of cataracts, wet age-related macular degeneration (w-AMD), diabetic retinopathy, and disorders of the optic nerve and visual pathways. Conversely, smoking may be associated with a decreased risk of myopia [100].

The meta-analysis provides robust evidence that smoking substantially increases the risk of multiple ocular disorders, including age-related macular degeneration, cataract, diabetic retinopathy, and dry eye disease. By synthesizing data from numerous meta-analyses, the study demonstrates both the consistency and magnitude of smoking-related harm to ocular structures, highlighting clear dose–response relationships and stronger effects among current smokers compared with former smokers. These findings emphasize that smoking is a major, preventable risk factor for vision impairment and underscore the importance of integrating ocular health considerations into public health tobacco-control strategies [101].

### 6.9. Therapeutic Implications

Recent advances in COPD treatment strongly support the immune-inflammatory mechanisms discussed here. Notably, the 2024 FDA approval of dupilumab marks the first biologic therapy for COPD, targeting IL-4 and IL-13 signaling in type 2 inflammation. Phase 3 trials showed approximately a 30% reduction in exacerbations, especially in patients with eosinophilic COPD, highlighting the role of adaptive immune skewing and cytokine-driven inflammation in specific COPD endotypes [102,103]. Similarly, the approval of ensifentrine, a dual phosphodiesterase-3/4 inhibitor, introduces a new non-steroidal anti-inflammatory and bronchodilator approach for COPD management, emphasizing the therapeutic potential of targeting intracellular signaling pathways that influence inflammation, airway smooth muscle tone, and epithelial function [104]. Together, these therapies represent a shift toward mechanism-based, precision treatment strategies that align with the immunopathogenic framework detailed in this review.

Beyond airway-focused interventions, emerging evidence links the gut–lung axis as an important and potentially modifiable pathway in COPD. Recent human and experimental studies show that CS causes gut microbiota imbalance, decreases short-chain fatty acid levels, and disrupts mucosal immune balance, with circulating propionic acid levels positively associated with lung function [105]. Notably, smoke-induced gut–lung dysbiosis and related inflammatory changes seem to last long after stopping exposure, indicating a role for microbiota-related immune memory in disease persistence [106]. These findings pave the way for new treatments, including dietary fiber supplements, probiotics or symbiotics, and microbiota-targeted therapies like fecal microbiota transplantation, which may enhance pharmacologic treatments by influencing systemic and mucosal immune responses.

## 7. Conclusions

CS triggers a complex, multi-layered immune and inflammatory response that significantly impacts human health at both local and systemic levels. The process starts with oxidative stress and molecular damage, including the production of ROS, lipid peroxidation, and DNA damage, which undermine cellular integrity and initiate early immune responses. These initial injuries activate both the innate immune system, through mechanisms such as neutrophil and macrophage activation, pattern recognition receptor (PRR) signaling, and inflammasome engagement, as well as the adaptive immune system, involving T-cell dysregulation and altered B-cell responses. The interaction of these immune responses results in a persistent, low-grade systemic inflammation that plays a central role in chronic disease development. Mechanistically, smoking induces epigenetic changes, including DNA methylation, histone modifications, and microRNA dysregulation, further worsening immune dysfunction and maintaining inflammatory signaling over time. These overlapping pathways drive the development of a broad range of disorders: COPD through airway remodeling and alveolar destruction; cardiovascular diseases via endothelial dysfunction, atherosclerosis, and prothrombotic conditions; metabolic disorders like insulin resistance and type 2 diabetes, driven by chronic inflammation and oxidative damage; increased vulnerability to infections owing to impaired mucosal defenses and suppressed innate immunity; and cancer, especially lung cancer, through genomic instability, mutagenesis, and compromised immune surveillance.

Importantly, CS remains one of the most potent and modifiable risk factors for these chronic conditions. Its effects are not limited to any single organ system but involve a system-wide pathological network where oxidative stress, immune dysregulation, epigenetic changes, and chronic inflammation intersect. These mechanisms collectively explain the wide range of smoking-related health issues and emphasize the importance of smoking cessation as a key preventative measure. Moreover, understanding these interconnected pathways offers opportunities for targeted therapies, including antioxidants, immunomodulators, and epigenetic agents, to reduce the burden of smoking-induced diseases.

## Figures and Tables

**Table 1:** Tobacco-specific nitrosamines (TSNAs).

TSNAs	Structure	Effects
**N′-nitrosonornicotine (NNN)**	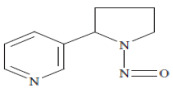	Esophageal cancer, Nasal and oral cavity cancer
**4-(methylnitrosamino)-1-(3-pyridyl)-1-butanone (NNK)**	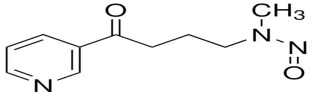	Lung cancer, Pancreas cancer, Liver cancer, Nasal and oral cavity cancer
**4-(methylnitrosamino)-1-(3-pyridyl)-1-butanol (NNAL)**	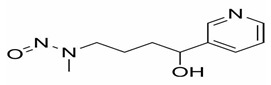	Lung cancer, Pancreas cancer, Liver cancer, Nasal and oral cavity cancer
**N′-nitrosoanabasine (NAB)**	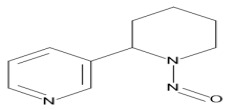	Esophageal cancer, Nasal cavity cancer
**N′-nitrosoanatabine (NAT)**	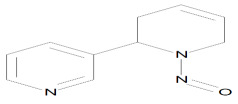	Esophageal cancer, Nasal cavity cancer

## Data Availability

No new data were created or analyzed in this study. Data sharing is not applicable to this article.
